# Supplementary material for: Yeast9: a consensus genome-scale metabolic model for S. cerevisiae curated by the community
Source: Mol Syst Biol. 2024 Aug 12;20(10):1134–50. doi: 10.1038/s44320-024-00060-7 (PMC11450192; doi:10.1038/s44320-024-00060-7)
Supplement: Supplementary file 11 — Expanded View Figures [file 44320_2024_60_MOESM11_ESM.pdf]

Expanded View Figures

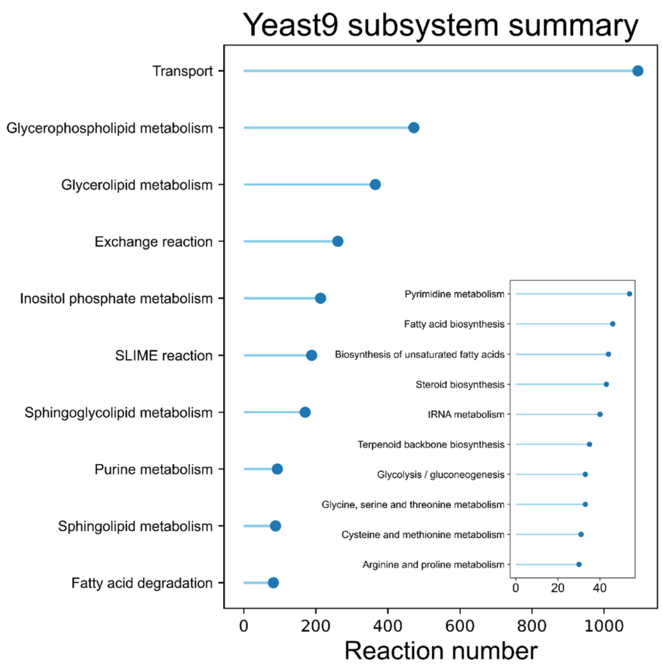

Figure EV1. Reaction subsystems distribution of yeast-GEM.

Top 20 reaction subsystems in yeast-GEM.

© The Author(s)

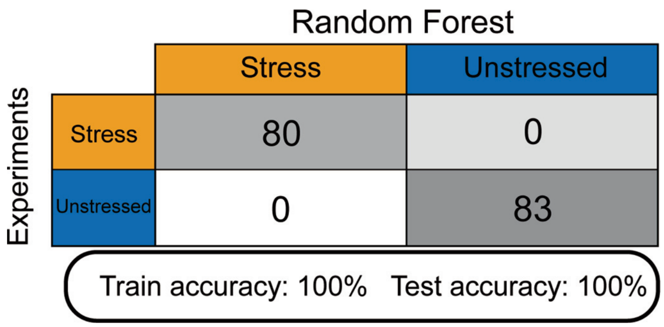

Figure EV2. Random Forest classifier prediction performance using transcriptomic data.

The Random Forest classifier has 100% accuracy in classifying the single cell from high osmotic stress and normal conditions using transcriptomic data.

 © The Author(s)

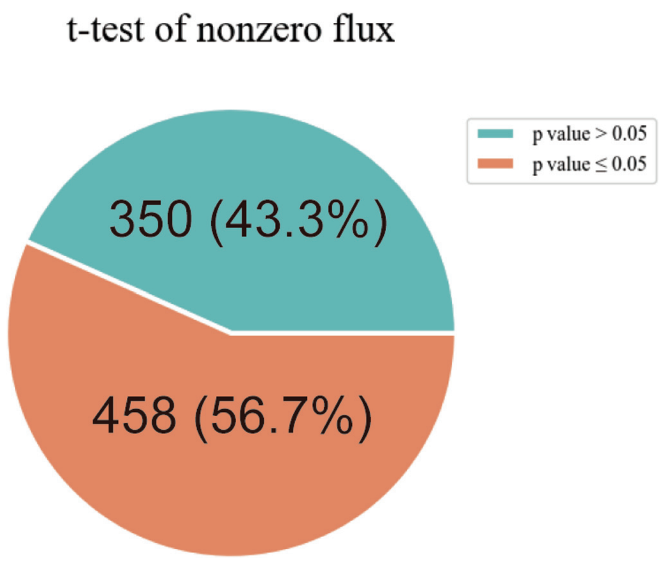

Figure EV3. Significance analysis of reactions' flux.

t test of active flux between salt stress condition and unstress condition.
